# Prognostic Significance of Left Ventricular Mass Index and Renal Function Decline Rate in Chronic Kidney Disease G3 and G4

**DOI:** 10.1038/srep42578

**Published:** 2017-02-14

**Authors:** Jiun-Chi Huang, Szu-Chia Chen, Yi-Chun Tsai, I-Ching Kuo, Yi-Wen Chiu, Jer-Ming Chang, Shang-Jyh Hwang, Hung-Chun Chen

**Affiliations:** 1 Graduate Institute of Clinical Medicine, College of Medicine, Kaohsiung Medical University, Kaohsiung, Taiwan; 2 Division of Nephrology, Department of Internal Medicine, Kaohsiung Medical University Hospital, Kaohsiung Medical University, Kaohsiung, Taiwan; 3 Department of Internal Medicine, Kaohsiung Municipal Hsiao-Kang Hospital, Kaohsiung Medical University, Kaohsiung, Taiwan; 4 Faculty of Medicine, College of Medicine, Kaohsiung Medical University, Kaohsiung, Taiwan; 5 Department of Internal Medicine, Kaohsiung Municipal Ta-Tung Hospital, Kaohsiung Medical University, Kaohsiung, Taiwan; 6 Faculty of Renal Care, College of Medicine, Kaohsiung Medical University, Kaohsiung, Taiwan; 7 Department of Internal Medicine, Kaohsiung Municipal Cijin Hospital, Kaohsiung Medical University, Kaohsiung, Taiwan; 8 Institute of Population Sciences, National Health Research Institutes, Miaoli, Taiwan

## Abstract

The effect of left ventricular mass index (LVMI) and estimated glomerular filtration rate (eGFR) decline rate on outcome prediction in patients with chronic kidney disease (CKD) remains unclear. We included 306 CKD G3 and G4 patients with LVMI assessed through echocardiography. Rapid decline in renal function was defined as the eGFR slope <−3 mL/min/1.73 m^2^/year. Patients were stratified into four groups using sex-specific median values of LVMI and rapid eGFR decline. The composite outcome was progression to maintenance dialysis or death. 32 patients had the composite outcome during a median follow-up of 2.7 years. In multivariate Cox analysis, compared with patients with non-rapid eGFR decline and lower LVMI, those with non-rapid eGFR decline and higher LVMI (hazard ratio [HR]: 5.908, 95% confidence interval [CI] = 1.304–26.780), rapid eGFR decline and lower LVMI (HR: 12.737, 95% CI = 2.297–70.636), and rapid eGFR decline and higher LVMI (HR: 15.249, 95% CI = 3.365–69.097) had an increased risk of progression to adverse outcomes. LVMI and eGFR decline synergistically effect the prognostic implications in CKD G3 and G4 patients.

Chronic kidney disease (CKD) is an emerging major public health issue worldwide. Some patients with CKD experience a progressive loss of kidney function and progression to end-stage renal disease. Furthermore, many patients with CKD may not survive to reach the maintenance dialysis stage^1^,^2^. With declines in kidney function, cardiovascular disease (CVD) remains the leading cause of death^3^. Prognostic studies, mostly based on the baseline level of the estimated glomerular filtration rate (eGFR), have shown that decreased kidney function is associated with increased mortality and morbidity^3^,^4^. However, several recent reports have highlighted the impact of dynamic changes in the eGFR on the risk of death^5^,^6^,^7^,^8^. The renal function decline rate may be a valuable prognostic indicator in patients with CKD.

CKD considerably increases the risk of CVD. Given that CKD and CVD share many of the same risk factors, abnormal cardiac structure and function is one of principal predictors of adverse clinical outcomes^9^. Left ventricular hypertrophy (LVH) is highly prevalent in CKD patients and is associated with the risk of mortality and unfavorable prognosis^10^,^11^,^12^. In addition, CKD patients have progressively increasing left ventricular (LV) mass with decreased renal function^10^. The effect of the interaction between the LVMI and eGFR decline rate on the prediction of the renal outcomes in patients with CKD remains unclear.

Therefore, the present study investigates whether the combination of the LVMI and eGFR decline rate is associated with the composite outcome of disease progression to maintenance dialysis or death in CKD G3 and G4 patients.

## Results

On the basis of the decline rate in renal function (i.e., non-rapid or rapid eGFR decline) and sex-specific median value of the LVMI (male: 129.4 g/m^2^ and female: 118.9 g/m^2^), 306 patients with CKD G3 and G4 were stratified into four groups. Table 1 lists the baseline clinical characteristics of study patients.

Of the 306 patients, 217 (70.9%) were men, and 174 (56.9%) had diabetes. The mean age was 67.1 ± 11.6 years and the mean baseline eGFR was 33.4 ± 10.8 mL/min/1.73 m^2^. During the follow-up period, the median number of serum creatinine measurements was 8 (range, 5–11). The median eGFR slope value was −0.9 (range, −2.5 to 0.3) mL/min/1.73 m^2^/year. Patients with rapid eGFR decline and a higher LVMI were more likely to have lower baseline eGFR and lower serum albumin levels, as well as higher systolic blood pressure, uric acid levels, and proteinuria prevalence, and a higher number of serum creatinine measurements compared with those with non-rapid eGFR decline and a lower LVMI.

### Risk factors for progression to the composite outcome

**Figure 1:**
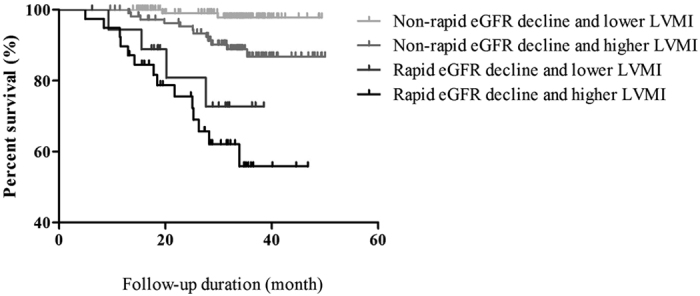
Kaplan–Meier curves for composite outcome-free survival according to eGFR decline rate and LVMI (log rank p < 0.001). Patients with rapid eGFR decline and a higher LVMI had the lowest probability of composite outcome-free survival.

In total, 18 patients died (9 from CVD, 8 from infection or sepsis, and 1 from gastrointestinal bleeding) and 14 progressed to maintenance dialysis during a median follow-up of 32.0 (range, 22.5–36.3) months. Figure 1 presents the Kaplan–Meier curves of composite outcome-free survival among the four groups of study patients. Patients with rapid eGFR decline and a higher LVMI had the lowest probability of composite outcome-free survival (log-rank p < 0.001).

**Figure 2:**
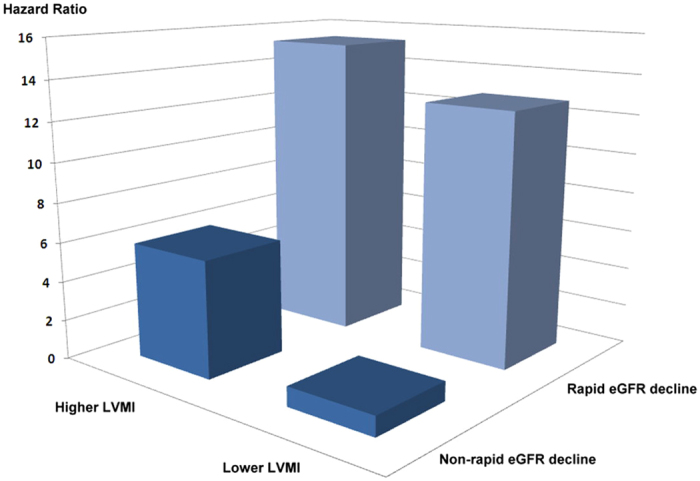
Synergistic effect of eGFR decline rate and LVMI on the prediction of the composite outcome.

Table 2 shows a Cox proportional hazards analysis of progression to the composite outcome. In order to avoid over-fitted adjusted analysis, all the variables in Table 1 were tested by univariate analysis and those variables with p-value less than 0.05 were selected into multivariable stepwise analysis (Supplementary Table S1). Rapid eGFR decline and higher LVMI, baseline eGFR, serum uric acid, and total calcium levels were significantly associated with maintenance dialysis or death. These four variables were included in the final adjusted model. The multivariate Cox analysis showed that compared with patients with non-rapid eGFR decline and a lower LVMI, those with non-rapid eGFR decline and a higher LVMI (hazard ratio [HR]: 5.908; 95% CI = 1.304–26.780, p-value = 0.021), rapid eGFR decline and a lower LVMI (HR: 12.737; 95% CI = 2.297–70.636, p-value = 0.004), and rapid eGFR decline and a higher LVMI (HR: 15.249; 95% CI = 3.365–69.097, p-value < 0.001) were significantly associated with an increased risk of progression to the composite outcome. In addition, a low baseline eGFR (HR: 0.937; 95% CI = 0.895–0.981, p-value = 0.005), high serum uric acid level (HR: 1.206; 95% CI = 1.026–1.417; p-value = 0.023), and low serum total calcium level (HR: 0.555; 95% CI = 0.352–0.875; p-value = 0.011) were significantly associated with an increased risk of progression to maintenance dialysis or death in the multivariate Cox analysis. A synergistic effect of the eGFR decline rate and LVMI on the prediction of renal outcomes was observed (Fig. 2).

### Effect of adding the LVMI on the prediction of the composite outcome

**Figure 3:**
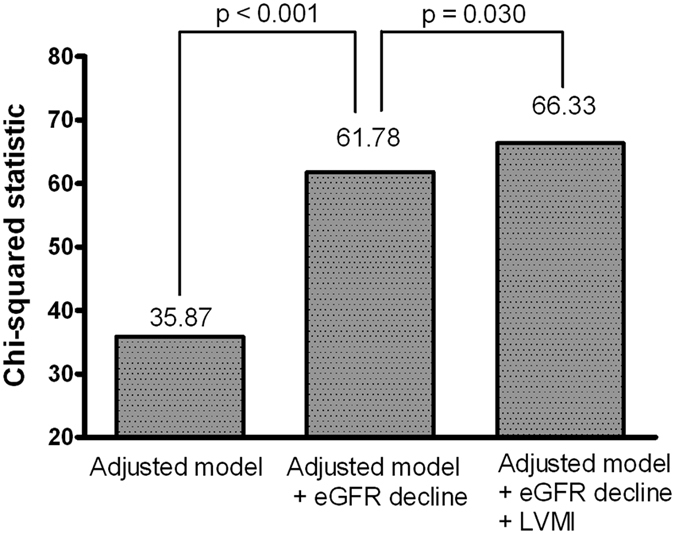
Addition of LVMI to the Cox model containing clinical variables and eGFR decline rate improved the prediction of the composite outcome.

We analyzed the effect of adding the LVMI on the prediction of the composite outcome (Fig. 3). The adjusted model included the baseline eGFR, uric acid, and total calcium. After adding the eGFR decline rate to the adjusted model, an incremental benefit was observed in the prediction of the composite outcome (χ^2^ = 35.87 to 61.78, p-value < 0.001). Furthermore, the addition of the LVMI to the model containing adjusted model and the eGFR decline rate resulted in further significant improvement in the prediction of the composite outcome (χ^2^ = 61.78 to 66.33, p-value = 0.030).

### Risk factors for progression to maintenance dialysis and death

We also identified the risk factors for the each two outcomes. High LVMI, fast decline in eGFR, and low baseline eGFR were associated with commencing dialysis (Supplementary Table S2). High LVMI, low serum albumin, high glycated hemoglobin, and high uric acid levels were associated with death in multivariate adjusted model (Supplementary Table S3).

## Discussion

This study investigated the effect of the interaction between the LVMI and renal function decline rate on the adverse outcomes in patients with CKD G3–4 over an observation period of 2.7 years. Our results showed the synergistic effect of the LVMI and eGFR decline rate on the prediction of an unfavorable composite outcome, including maintenance dialysis or death in patients with CKD G3 and G4.

CKD and CVD share certain risk factors, such as hypertension, vascular stiffness, and endothelial dysfunction^13^. Prior studies have shown that a higher prevalence of LVH in patients with CKD than in the general population^10^. Moreover, a gradual increment in the LVMI has been observed with the progression of CKD^10^,^14^. The pathogenesis mediated increased LVMI in patients with CKD is multifactorial in origin. Changes in the vascular wall, such as increased vascular stiffness and calcification, occur with advancing age. Stiffness of major vessels contributes to increased peripheral resistance, worsened hypertension and elevated pulse pressure, which in turn augment LV mass^15^. Moreover, proteinuria appears to be a marker of underlying inflammation, oxidative stress, and endothelial dysfunction^16^. Interplay of these factors could result in vascular injury and myocardial hypertrophy and fibrosis, leading to renal and cardiac involvement.

The well-known risk factors, such as the baseline eGFR, proteinuria, and arterial hypertension, contribute to the development of unfavorable outcomes in CKD. However, the mechanisms involved in CKD progression are complicated, and these traditional elements cannot explain the entire risk of CKD progression. Paoletti *et al*.^11^ recognized a higher LVMI as a strong prognostic indicator of disease progression to maintenance dialysis or death in non-diabetic patients with CKD G3 to G4. Similarly, we observed that patients with a higher LVMI had an elevated risk of adverse renal outcomes, irrespective of the renal function decline rate. Moreover, rapid eGFR decline and a higher LVMI had a synergistic effect on the prediction of the composite outcome. The addition of the LVMI to the model containing the rate of eGFR decline provided a more satisfactory predicting performance for unfavorable prognosis in patients with CKD G3 to G4. Furthermore, our results may raise awareness regarding the importance of the eGFR slope and its potential practical benefits in risk stratification for patients with CKD. Taken together, the interaction of functional and structural derangements in the heart and kidney amplifies the progression of CKD and is associated with unfavorable renal outcomes.

Based on the competing risk between maintenance dialysis and death in CKD patients, we performed competing risk analysis and the adjusted HR of death in those with rapid eGFR decline and higher LVMI compared to those with non-rapid eGFR decline and lower LVMI was 11.027 (95% CI: 2.220–54.779, Supplementary Table S4). We further analyzed the influence of competing risk of death on maintenance dialysis. However, the coefficient did not converge and statistics could not be completed. In our study group, none of patients with non-rapid eGFR decline progressed to maintenance dialysis during follow-up period. The median values of eGFR slope in patients with non-rapid eGFR decline and lower LVMI and in those with non-rapid eGFR decline and higher LVMI were −0.4 and −0.5 mL/min/1.73 m^2^/year, respectively. The observation period is not long enough to have renal progression to maintenance dialysis in these patients. Besides, the relatively small number of events (18 of death and 14 of maintenance dialysis) might be one of the reasons of incomplete analysis. Because the study included only patients with G3 to G4 CKD, our results might not be generalizable to all CKD populations. Future work that includes more patients with different stages of CKD is necessary for more clearly elucidating the relationship between the LVMI and renal function decline rate. Additionally, cardiac magnetic resonance imaging (CMRI) is the gold standard tool for assessing LV dimensions as well as the degree of cardiac fibrosis in CKD patients^9^. Compared with CMRI, echocardiography is less costly and more practical and easily available. Although echocardiography may overestimate LV mass relative to CMRI in maintenance hemodialysis patients^17^, it is widely used for evaluating LV geometry. Lastly, proteinuria was assessed using dipsticks rather than urine protein-to-creatinine ratio, which might not be a precise quantitative measurement of proteinuria.

In conclusion, our study demonstrated that a higher LVMI and rapid eGFR decline are associated with unfavorable renal outcomes in CKD G3 and G4 patients. Moreover, the LVMI and eGFR decline rate synergistically affect the prediction of poor outcomes. Our findings may reinforce the major role of echocardiography in the detection of cardiac structural abnormalities and the trajectories of eGFR decline in this high-risk population. Future studies and therapy that aim at halting LV remodeling and progression of CKD are warranted.

## Methods

### Participants and study design

**Figure 4:**
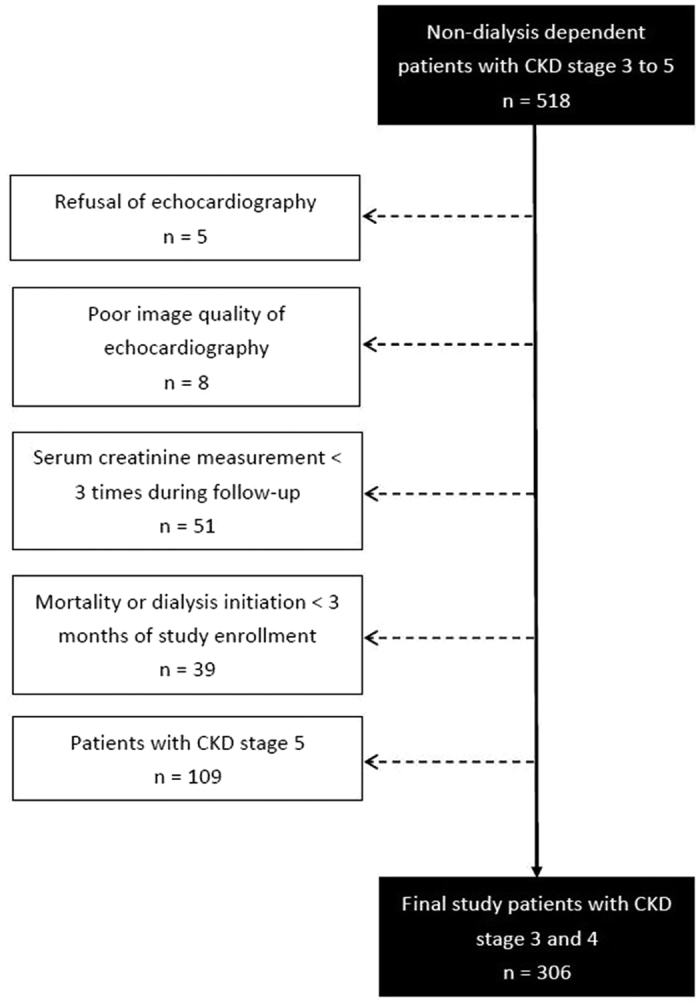
Flowchart of participants analyzed in this study.

This was a retrospective and observational study at a regional hospital in Taiwan. 518 dialysis independent patients with CKD G3 to G5 were enrolled from the outpatient department of internal medicine during January 2007–May 2010. Kidney function was quantified as the eGFR calculated by the equation of the 4-variable Modification of Diet in Renal Disease (MDRD) Study^18^. According to the Kidney Disease Improving Global Outcomes guidelines^19^, on the basis of the participants’ baseline eGFR, CKD stages were stratified as G3 (eGFR = 30–59 mL/min/1.73 m^2^), G4 (eGFR = 15–29 mL/min/1.73 m^2^), and G5 (eGFR <15 mL/min/1.73 m^2^) based on the participants’ baseline eGFR. However, we excluded 5 patients who refused to undergo echocardiography, 8 patients with poor quality of echocardiographic image visualization, and 51 patients with less than three available serum creatinine measurements during follow-up. We also excluded 9 patients who died and 30 who required dialysis therapy within 3 months after study enrollment to avoid incomplete observation of decline in renal function. Moreover, 109 patients with CKD G5 were excluded because of complex factors influencing outcomes and dialysis commencement decisions^20^,^21^. Finally, 306 patients with CKD G3 and G4 were included in this study (Fig. 4). This study was approved by the Institutional Review Board at Kaohsiung Medical University Hospital. All clinical investigations were conducted in accordance with the principles of the Declaration of Helsinki. Informed consent was obtained from all patients.

### Echocardiography

To assess cardiac structure and function, echocardiographic examination was performed using VIVID 7 (General Electric Medical Systems, Horten, Norway) by two experienced cardiologists blinded to the clinical data of the study patients. Two-dimensional and two-dimensionally guided M-mode images were recorded from the standardized views according to the American Society of Echocardiography recommendations^22^. Interventricular septal wall thickness in diastole (IVSTd), left ventricular internal diameter in diastole (LVIDd), and left ventricular posterior wall thickness in diastole (LVPWTd) were measured in the left parasternal long-axis view. LV mass was calculated using the Devereux-modified method^23^. LV mass (in grams) = 1.04 × [(IVSTd + LVIDd + LVPWTd)^3^ − LVIDd^3^] − 13.6. LVMI was calculated as LV mass divided by body surface area.

### Demographic, medical, and laboratory data collection

We collected baseline variables of the study patients at the baseline visit, including demographic characteristics (age and sex), comorbidities, such as diabetes, hypertension, coronary artery disease, cerebrovascular disease, smoking habit (ever *vs.* never), examination findings (body mass index [BMI], and systolic and diastolic blood pressure), renal function status (baseline eGFR and proteinuria), and medications (angiotensin-converting enzyme [ACE] inhibitors or angiotensin receptor blockers [ARBs], beta-blockers, aspirin, and statins). Demographic characteristics and medical history were obtained through the baseline medical chart records and by reviewing physicians’ charts and interviews with the study patients. BMI was defined as body weight (in kilograms) divided by the squared height (in meters). Blood pressure was measured using a manual sphygmomanometer at the baseline visit with participants in the sitting position after 5 minutes of rest. Overnight fasting blood samples were measured for laboratory data. The compensated Jaffé method was used for measurement of serum creatinine. Proteinuria was detected by dipsticks (Hema-Combistix, Bayer Diagnostics). Proteinuria of ≥1+ was defined as a positive result. Urine and blood samples were measured within one month of study enrollment.

### Assessment of decline rate in renal function

The rate of renal function decline was determined according to the slope, defined as the regression coefficient between eGFR and time, representing the change in the eGFR over time. All available eGFR values from the echocardiographic examination until the beginning of maintenance dialysis, death, or the end of the observation period were included for calculation of the eGFR slope. At least three eGFR values were needed to calculate the eGFR slope using regression coefficient between eGFR and time. Rapid decline in renal function was defined as the eGFR slope <−3 mL/min/1.73 m^2^/year^8^,^24^.

### Composite outcome

The composite outcome was defined as either disease progression to maintenance dialysis treatment or death. The patients were censored at death, last contact, or the end of observation in March 2011. Patients dying after maintenance dialysis were excluded. The patients were contacted at outpatient clinics at 3-month intervals to ascertain their clinical status. Maintenance dialysis was confirmed by reviewing medical charts or catastrophic illness certificates issued by the Bureau of National Health Insurance in Taiwan.

### Statistical analysis

Data presented as percentages for categorical variables were analyzed using the *x*^2^ test. Continuous normally distributed variables are presented as the means ± standard deviations, or median (25^th^–75^th^ percentile) for the triglycerides level, intact parathyroid hormone level, and number of serum creatinine measurements. The study participants were stratified into four groups according to the decline rate of kidney function (non-rapid or rapid eGFR decline) and sex-specific median value of the LVMI. Multiple comparisons among the study groups were performed using one-way analysis of variance followed by a post hoc test adjusted with a Bonferroni correction. The survival curve for the composite outcome was illustrated using the Kaplan–Meier method and the four groups were compared using the log-rank test. Time to the composite outcome and covariates were analyzed using a Cox proportional hazards model. Covariates were selected into multivariate Cox models if their p-value was <0.05 in univariate analysis. A p-value of <0.05 was considered statistically significant. Statistical analyses were conducted using SPSS 22.0.0.0 (SPSS Inc., Chicago, IL) and SAS 9.2 (SAS Institute, Cary, NC) for Windows.

## Additional Information

**How to cite this article:** Huang, J.-C. *et al*. Prognostic Significance of Left Ventricular Mass Index and Renal Function Decline Rate in Chronic Kidney Disease G3 and G4. *Sci. Rep.* **7**, 42578; doi: 10.1038/srep42578 (2017).

**Publisher's note:** Springer Nature remains neutral with regard to jurisdictional claims in published maps and institutional affiliations.

## Supplementary Material

Supplementary Tables

## Figures and Tables

**Table 1:** Baseline clinical characteristics of study patients.

Characteristic	All patients (n = 306)	Non-rapid eGFR decline		Rapid eGFR decline		p-value
		Lower LVMI (n = 134)	Higher LVMI (n = 114)	Lower LVMI (n = 19)	Higher LVMI (n = 39)	
Demographic and medical history						
Age (years)	67.1 ± 11.6	65.6 ± 12.2	67.7 ± 11.3	67.2 ± 11.5	70.5 ± 9.4	0.117
Men (%)	70.9	70.9	69.3	68.4	76.9	0.831
Smoking habits (%)	31.4	31.3	33.3	10.5	35.9	0.222
Diabetes mellitus (%)	56.9	51.5	57.0	84.2^*^	61.5	0.052
Hypertension (%)	78.1	75.4	79.8	73.7	84.6	0.583
Coronary artery disease (%)	10.8	6.0	12.3	15.8	20.5	0.05
Cerebrovascular disease (%)	13.4	11.9	12.3	21.1	17.9	0.571
Renal function status						
Baseline eGFR (mL/min/1.73 m^2^)	33.4 ± 10.8	35.1 ± 10.8	33.4 ± 10.7	30.1 ± 8.5	29.6 ± 11.3^*^	0.02
eGFR slope (mL/min/1.73 m^2^/year)	−0.9 (−2.5–0.3)	−0.4 (−1.6–0.7)	−0.5 (−1.6–0.6)	−4.8 (−7.1–−3.7)^*,†^	−5.1 (−6.5–−3.9)^*,†^	<0.001
Echocardiographic measurement						
LVMI (g/m^2^)	132.0 ± 45.7	99.0 ± 19.0	164.7 ± 39.5^*^	95.2 ± 21.0^†^	167.6 ± 40.0^*,#^	<0.001
Examination findings						
Systolic BP (mmHg)	138.5 ± 19.2	135.4 ± 17.9	140.5 ± 19.0	135.8 ± 22.9	144.9 ± 20.9^*^	0.029
Diastolic BP (mmHg)	79.7 ± 12.1	78.8 ± 10.3	80.4 ± 13.1	76.6 ± 14.1	82.3 ± 13.9	0.269
BMI (kg/m^2^)	25.6 ± 3.8	25.3 ± 4.1	25.8 ± 3.6	25.1 ± 4.0	26.2 ± 3.2	0.506
Laboratory data						
Albumin (g/dL)	4.1 ± 0.3	4.2 ± 0.3	4.1 ± 0.3	4.0 ± 0.3	4.0 ± 0.3^*^	0.002
Hemoglobin (g/dL)	12.5 ± 2.0	12.6 ± 1.9	12.7 ± 2.1	12.2 ± 1.8	11.8 ± 1.9	0.098
Total cholesterol (mg/dL)	194.2 ± 43.4	187.9 ± 37.8	197.5 ± 45.2	206.2 ± 57.2	200.7 ± 47.6	0.128
Triglycerides (mg/dL)	136 (96–202)	133 (95–190)	139 (95–208)	177 (119–229)	134 (98–210)	0.431
Uric acid (mg/dL)	8.0 ± 2.1	7.6 ± 1.9	8.1 ± 2.1	8.6 ± 2.9	8.7 ± 2.0^*^	0.017
Total calcium (mg/dL)	9.7 ± 0.7	9.6 ± 0.6	9.7 ± 0.6	9.7 ± 1.1	9.5 ± 0.9	0.364
Phosphorus (mg/dL)	3.7 ± 0.6	3.6 ± 0.6	3.7 ± 0.5	3.9 ± 0.9	3.8 ± 0.6	0.309
iPTH (pg/mL)	64 (55–75)	64 (54–73)	63 (53–76)	71 (56–90)	67 (58–79)	0.037
HbA1C (%)	6.9 ± 1.6	6.6 ± 1.2	6.9 ± 1.6	8.0 ± 2.8^*^	7.4 ± 1.8	0.001
Proteinuria						
0 (%)	47.1	53.7	49.1	36.8	23.1^*,†^	0.006
1+ (%)	20.6	26.1	21.1	5.3	7.7	0.026
>1+ (%)	32.4	20.1	29.8	57.9^*^	69.2^*,†^	<0.001
Numbers of creatinine measurement	8 (5–11)	7 (4–9)	9 (7–12)^*^	7 (4–11)	8 (6–16)^*^	0.002
Medications						
Aspirin (%)	26.8	21.6	33.3	15.8	30.8	0.152
ACE inhibitors and/or ARBs (%)	82.7	81.3	87.7	73.7	76.9	0.293
Beta-blockers (%)	28.4	20.1	33.3	36.8	38.5	0.041
Statins (%)	27.5	26.1	28.9	31.6	25.6	0.909

Abbreviations: eGFR, estimated glomerular filtration rate; LVMI, left ventricular mass index; LV, left ventricular; BP, blood pressure; BMI, body mass index; iPTH, intact parathyroid hormone; HbA1C, glycated hemoglobin; ACE, angiotensin-converting enzyme; ARBs, angiotensin receptor blockers.

^*^p < 0.05 in comparison with non-rapid eGFR decline and lower LVMI.

^†^*p* < 0.05 in comparison with non-rapid eGFR decline and higher LVMI.

^#^*p* < 0.05 in comparison with rapid eGFR decline and lower LVMI.

**Table 2:** Predictors of progression to the composite outcome using Cox proportional hazards model.

Parameter	Univariate		Multivariate	
	HR (95% CI)	p-value	HR (95% CI)	p-value
Study groups				
Non-rapid eGFR decline and lower LVMI	Reference	—	Reference	—
Non-rapid eGFR decline and higher LVMI	6.378 (1.427–28.507)	0.015	5.908 (1.304–26.780)	0.021
Rapid eGFR decline and lower LVMI	17.926 (3.279–97.985)	0.001	12.737 (2.297–70.636)	0.004
Rapid eGFR decline and higher LVMI	29.941 (6.799–131.864)	<0.001	15.249 (3.365–69.097)	<0.001
Baseline eGFR (per 1 mL/min/1.73 m^2^)	0.913 (0.874–0.953)	<0.001	0.937 (0.895–0.981)	0.005
Uric acid (per 1 mg/dL)	1.358 (1.161–1.588)	<0.001	1.206 (1.026–1.417)	0.023
Total calcium (per 1 mg/dL)	0.483 (0.304–0.768)	0.002	0.555 (0.352–0.875)	0.011

Data are presented as hazard ratio (HR) and 95% confidence interval (CI).

Abbreviations are the same as Table 1.
