# Supplementary material for: Prognostic Significance of Left Ventricular Mass Index and Renal Function Decline Rate in Chronic Kidney Disease G3 and G4
Source: Sci Rep. 2017 Feb 14;7:42578. doi: 10.1038/srep42578 (PMC5307355; doi:10.1038/srep42578)
Supplement: Supplementary Tables [file srep42578-s1.pdf]

Supplementary Information

Prognostic Significance of Left Ventricular Mass Index and Renal Function Decline Rate in Chronic Kidney Disease G3 and G4

Jiun-Chi Huang, M.D., Szu-Chia Chen, M.D., Yi-Chun Tsai, M.D., I-Ching Kuo, M.D., Yi-Wen Chiu, M.D., Jer-Ming Chang, M.D., Ph.D., Shang-Jyh Hwang, M.D., Hung-Chun Chen, M.D., Ph.D.

Supplementary Table S1. Predictors of progression to the composite outcome using univariate and multivariate forward adjusted Cox models

Parameter	Univariate		Multivariate (Forward)	
	HR (95% CI)	p-value	HR (95% CI)	p-value
Study groups				
Non-rapid eGFR decline and lower LVMI	Reference	−	Reference	−
Non-rapid eGFR decline and higher LVMI	6.378 (1.427−28.507)	0.015	5.783 (1.278−26.165)	0.023
Rapid eGFR decline and lower LVMI	17.926 (3.279−97.985)	0.001	8.179 (1.335−50.127)	0.023
Rapid eGFR decline and higher LVMI	29.941 (6.799−131.864)	< 0.001	17.949 (3.963−81.291)	< 0.001
Age (per 1 year)	1.052 (1.014−1.091)	0.007	−	−
Men (*vs*. women)	1.494 (0.646−3.455)	0.348	−	−
Smoking history (ever *vs*. never)	1.959 (0.978−3.922)	0.058	−	−
Diabetes mellitus	2.013 (0.931−4.351)	0.075	−	−
Hypertension	1.499 (0.577−3.894)	0.406	−	−
Coronary artery disease	1.248 (0.438−3.559)	0.678	−	−
Cerebrovascular disease	1.225 (0.471−3.184)	0.677	−	−
Baseline eGFR (per 1 mL/min/1.73 m^2)	0.913 (0.874−0.953)	< 0.001	0.933 (0.892−0.976)	0.002
Systolic BP (per 1 mmHg)	1.019 (1.001−1.037)	0.040	−	−
Diastolic BP (per 1 mmHg)	0.993 (0.964−1.022)	0.637	−	−
BMI (per 1 kg/m^2)	0.968 (0.884−1.061)	0.493	−	−
Albumin (per 1 g/dL)	0.203 (0.089−0.464)	< 0.001	−	−
Hemoglobin (per 1 g/dL)	0.776 (0.649−0.929)	0.006	−	−
Total cholesterol (per 1 mg/dL)	1.001 (0.993−1.010)	0.736	−	−
Log-transformed triglycerides (per 1 log mg/dL)	0.840 (0.200−3.526)	0.812	−	−
HbA1C (per 1%)	1.175 (1.000−1.380)	0.050	−	−
Uric acid (per 1 mg/dL)	1.358 (1.161−1.588)	< 0.001	1.244 (1.045−1.480)	0.014
Total calcium (per 1 mg/dL)	0.483 (0.304−0.768)	0.002	0.548 (0.367−0.820)	0.003
Phosphorous (per 1 mg/dL)	1.787 (1.073−2.976)	0.026	−	−
Log-transformed iPTH (per 1 log pg/mL)	8.135 (2.680−24.692)	< 0.001	−	−
Proteinuira	2.228 (1.031−4.816)	0.042	−	−
Use of aspirin	1.995 (0.985−4.041)	0.055	−	−
Use of ACE inhibitors and/or ARBs	0.873 (0.359−2.123)	0.765	−	−
Use of beta-blockers	2.398 (1.197−4.803)	0.014	−	−
Use of statins	1.260 (0.597−2.662)	0.505	−	−

Abbreviations: eGFR, estimated glomerular filtration rate; LVMI, left ventricular mass index; BP, blood pressure; BMI, body mass index; iPTH, intact parathyroid hormone; HbA1C, glycated hemoglobin; ACE, angiotensin-converting enzyme; ARBs, angiotensin receptor blockers.

Supplementary Table S2. Predictors of progression to maintenance dialysis using univariate and multivariate forward adjusted Cox models

Parameter	Univariate		Multivariate (Forward)	
	HR (95% CI)	p-value	HR (95% CI)	p-value
LVMI (per 1 g/m^2)	1.011 (1.003−1.019)	0.008	1.011 (1.000−1.022)	0.041
eGFR slope (per 1 mL/min/1.73 m^2/year)	0.562 (0.458−0.689)	< 0.001	0.539 (0.376−0.773)	0.001
Age (per 1 year)	1.034 (0.982−1.088)	0.206	−	−
Men (*vs*. women)	1.527 (0.426−5.473)	0.516	−	−
Smoking history (ever *vs*. never)	1.670 (0.579−4.813)	0.342	−	−
Diabetes mellitus	1.416 (0.474−4.226)	0.533	−	−
Hypertension	1.658 (0.371−7.409)	0.508	−	−
Coronary artery disease	2.389 (0.666−8.567)	0.181	−	−
Cerebrovascular disease	0.507 (0.666−3.882)	0.513	−	−
Baseline eGFR (per 1 mL/min/1.73 m^2)	0.861 (0.791−0.936)	< 0.001	0.894 (0.801−0.998)	0.045
Systolic BP (per 1 mmHg)	1.031 (1.005−1.057)	0.018	−	−
Diastolic BP (per 1 mmHg)	1.010 (0.969−1.053)	0.643	−	−
BMI (per 1 kg/m^2)	1.048 (0.927−1.185)	0.456	−	−
Albumin (per 1 g/dL)	0.174 (0.053−0.577)	0.004	−	−
Hemoglobin (per 1 g/dL)	0.959 (0.679−1.353)	0.811	−	−
Total cholesterol (per 1 mg/dL)	0.996 (0.982−1.010)	0.564	−	−
Log-transformed triglycerides (per 1 log mg/dL)	1.275 (0.143−11.328)	0.828	−	−
HbA1C (per 1%)	0.959 (0.679−1.353)	0.811	−	−
Uric acid (per 1 mg/dL)	1.156 (0.897−1.491)	0.262	−	−
Total calcium (per 1 mg/dL)	0.476 (0.253−0.897)	0.022	−	−
Phosphorous (per 1 mg/dL)	2.487 (1.322−4.645)	0.004	−	−
Log-transformed iPTH (per 1 log pg/mL)	43.276 (9.729−192.503)	< 0.001	−	−
Proteinuira	5.259 (1.177−23.500)	0.030	−	−
Use of aspirin	1.166 (0.366−3.719)	0.795	−	−
Use of ACE inhibitors and/or ARBs	0.732 (0.204−2.627)	0.632	−	−
Use of beta-blockers	2.677 (0.938−7.631)	0.066	−	−
Use of statins	1.533 (0.514−4.577)	0.444	−	−

Abbreviations are the same as Supplementary Table S1.

Supplementary Table S3. Predictors of progression to death using univariate and multivariate forward adjusted Cox models

Parameter	Univariate		Multivariate (Forward)	
	HR (95% CI)	p-value	HR (95% CI)	p-value
LVMI (per 1 g/m^2)	1.009 (1.002−1.017)	0.016	1.010 (1.002−1.018)	0.015
eGFR slope (per 1 mL/min/1.73 m^2/year)	0.802 (0.678−0.948)	0.010	−	−
Age (per 1 year)	1.069 (1.014−1.126)	0.013	−	−
Men (*vs*. women)	1.448 (0.476−4.399)	0.514	−	−
Smoking history (ever *vs*. never)	2.072 (0.862−5.473)	0.100	−	−
Diabetes mellitus	2.721 (0.896−8.266)	0.078	−	−
Hypertension	1.357 (0.393−4.683)	0.630	−	−
Coronary artery disease	0.476 (0.063−3.576)	0.471	−	−
Cerebrovascular disease	1.911 (0.628−5.813)	0.254	−	−
Baseline eGFR (per 1 mL/min/1.73 m^2)	0.944 (0.897−0.993)	0.026	−	−
Systolic BP (per 1 mmHg)	1.007 (0.983−1.032)	0.571	−	−
Diastolic BP (per 1 mmHg)	0.977 (0.939−1.017)	0.258	−	−
BMI (per 1 kg/m^2)	0.898 (0.788−1.023)	0.105	−	−
Albumin (per 1 g/dL)	0.240 (0.076−0.758)	0.015	0.174 (0.049−0.623)	0.007
Hemoglobin (per 1 g/dL)	0.903 (0.714−1.143)	0.397	−	−
Total cholesterol (per 1 mg/dL)	1.005 (0.995−1.016)	0.319	−	−
Log-transformed triglycerides (per 1 log mg/dL)	0.599 (0.090−3.986)	0.596	−	−
HbA1C (per 1%)	1.294 (1.085−1.543)	0.004	1.225 (1.024−1.465)	0.026
Uric acid (per 1 mg/dL)	1.521 (1.240−1.865)	< 0.001	1.513 (1.215−1.884)	< 0.001
Total calcium (per 1 mg/dL)	0.610 (0.355−1.049)	0.074	−	−
Phosphorous (per 1 mg/dL)	1.168 (0.541−2.521)	0.693	−	−
Log-transformed iPTH (per 1 log pg/mL)	1.337 (0.238−7.510)	0.742	−	−
Proteinuira	1.336 (0.518−3.446)	0.550	−	−
Use of aspirin	2.978 (1.182−7.504)	0.021	−	−
Use of ACE inhibitors and/or ARBs	0.995 (0.288−3.438)	0.994	−	−
Use of beta-blockers	2.107 (0.832−5.340)	0.116	−	−
Use of statins	1.030 (0.367−2.889)	0.956	−	−

Abbreviations are the same as Supplementary Table S1.

Supplementary Table S4. The adjusted hazard ratios of death among study groups in competing risk analysis of maintenance dialysis

	HR (95% CI)	p-value
Study groups		
Non-rapid eGFR decline and lower LVMI	Reference	−
Non-rapid eGFR decline and higher LVMI	6.409 (1.441−28.501)	0.015
Rapid eGFR decline and lower LVMI	8.188 (1.134−59.147)	0.037
Rapid eGFR decline and higher LVMI	11.027 (2.220−54.779)	0.003

Abbreviations are the same as Supplementary Table S1.
